# Development and validation of a knowledge, attitude, and practice questionnaire regarding cardiovascular diseases in an Iranian general population

**DOI:** 10.1186/s12889-021-12135-3

**Published:** 2021-11-09

**Authors:** Fatemeh Koohi, Parisa Amiri, Yadollah Mehrabi, Mehrdad Karimi, Davood Khalili

**Affiliations:** 1 Obesity Research Center, Research Institute for Endocrine Sciences, Shahid Beheshti University of Medical Sciences, Tehran, Iran; 2 Research Center for Social Determinants of Health, Research Institute for Endocrine Sciences, Shahid Beheshti University of Medical Sciences, Tehran, Iran; 3 Department of Epidemiology, School of Public Health and Safety, Shahid Beheshti University of Medical Sciences, Tehran, Iran; 4 Prevention of Metabolic Disorders Research Center, Research Institute for Endocrine Sciences, Shahid Beheshti University of Medical Sciences, Tehran, Iran

**Keywords:** Cardiovascular diseases, Knowledge, Attitude, Practice, Validity, Questionnaire

## Abstract

**Background:**

Studies on knowledge, attitude, and practice (KAP) can be valuable for public health to help to develop targeted educational programs and assess the effectiveness of intervention programs. The purpose of this study was to develop and examine the validity and reliability of a questionnaire on knowledge, attitude, and practice (KAP) regarding cardiovascular diseases (CVDs), their risk factors, and symptoms among an Iranian general population.

**Methods:**

This cross-sectional study was conducted on an Iranian population older than 20 years referred to some of Tehran’s healthcare centers. An initial 62-item questionnaire was developed, and the face, content, and construct validities were assessed.

**Results:**

In all, 300 adults with a mean age (SD) of 39.79 (12.1) years participated in this study. Based on the results of the content validity, a questionnaire with 30 essential items was designed. Exploratory factor analysis suggested a four-factor subscale with 29 finalized items (CVD-KAP29), and acceptable goodness of fit indices was demonstrated by confirmatory factor analysis. The Cronbach’s alpha and McDonald’s ω coefficients were higher than 0.60 for all domains except the nutrition and smoking subscales.

**Conclusions:**

Results provided evidence of the validity of the CVD-KAP29 for KAP studies for cardiovascular diseases in the general population.

## Introduction

Cardiovascular diseases (CVDs) are the leading causes of death and disability worldwide [1], responsible for an estimated 17·9 million deaths each year; of these, more than 75% occur in low- and middle-income countries [2]. In Iran, according to GBD 2015, CVDs are the first cause of death, and DALYs, responsible for 46% of all deaths [3]. Considering that most CVD risk factors are preventable or controllable such as unhealthy diet, lack of physical activity, smoking, obesity, hypertension, diabetes, and dyslipidemia, and these diseases can be prevented mainly by developing more specific population-based prevention programs [4].

Although CVDs are multi-factorial events and several individual and socio-environmental factors influence their occurrence, it has been indicated that knowledge level in a health aspect led to changes in attitude. It gradually results in overt behavior change [5]. Therefore, it is evident that a vital prerequisite for changing health behaviors and lifestyles is increasing the knowledge on CVD and its modifiable risk factors [6, 7]. Finally, to assess the effectiveness of intervention programs, knowledge, attitude, and practice (KAP) surveys can be valuable [8] since it is vital for public health to help develop targeted educational programs [9]. For this, a valid and reliable instrument is needed to assess KAP.

To our knowledge, there is no reliable and validated questionnaire available on KAP for CVD, its risk factors, and symptoms for Iranian adults. In some previous studies, the psychometric properties of structured questionnaires were mainly assessed by internal consistency, without using statistical methods to test those tools’ construct validity and reliability [10, 11]. Moreover, most existing questionnaires have been developed for a specific country, while cultural, social, economic, and environmental status play a significant role in conducting behaviors to control cardiovascular risk factors [8, 12]; therefore, they might not apply to other countries. Hence, the purpose of this study was to develop, evaluated, and confirm the validity and reliability of the developed questionnaire on KAP regarding CVD, its risk factors, and symptoms among an Iranian general population.

## Material and methods

### Study design and participants

This cross-sectional study was conducted on an Iranian population older than 20 years of age referred to public healthcare centers in Tehran. A sample size of 300 was determined based on the rule of thumb of a subject to item ratio of 10:1 [13–15]. Because the socio-economic and education are different between urban and rural areas, a stratified sampling plan was considered to ensure representation regarding these areas. To do so, we stratified health care centers under the supervision of Shahid Beheshti University of Medical Sciences (SBMU) into urban and rural centers. A simple random sample of centers was taken from all 44 urban and rural health centers within each stratum (five urban and five rural centers). Then participants were selected using a convenience sampling from adults who were called for screening non-communicable diseases [16]. In total, 304 individuals were invited to participate in our study; 300 (98.7%) completed the questionnaire. Permission for the audio recording and written informed consent were taken from all participants before the interviews.

### The development process of CVD-KAP29

**Fig. 1:**
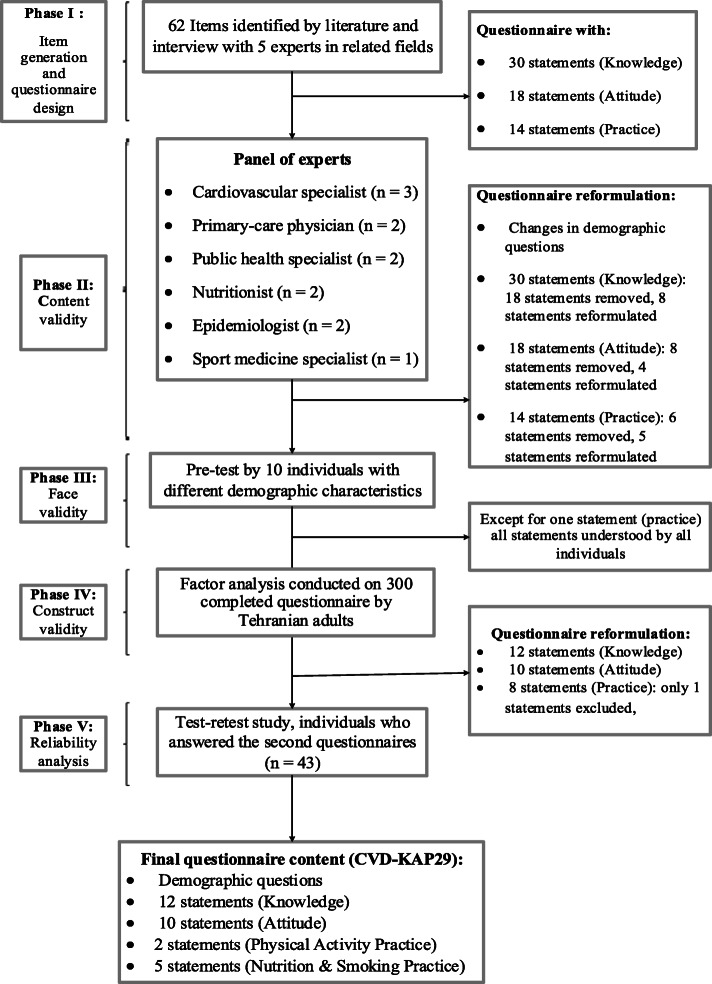
Quorum flow chart of the questionnaire development process

The CVD-KAP29 was developed based on five steps: item generation, assessing a face, content, construct validity, and reliability (Fig. 1).

### Phase I: item generation and questionnaire design

Relevant items were generated based on the results of both an intensive literature review and a semi-structured interview with five specialists in health education, epidemiology, cardiovascular disease, and nutrition. First, a literature search of previous studies regarding CVD knowledge, attitude, and practice was conducted to identify potential items for the study instrument through keywords of “cardiovascular disease”, “CVD”, “questionnaire”, “scale”, “knowledge”, “attitude”, “practice”, “behavior”, and “KAP” in English databases including PubMed, Scopus, and Google Scholar, as well as Persian equivalents of these terms, were used for searching in Persian databases including SID, IranDoc, and Magiran. Then we conducted ten interviews consisting of open-ended questions with experts who had agreed to participate in the study. The knowledge domain consisted of questions regarding the epidemiology of CVDs, their risk factors, and symptoms. We used the health belief model to design the attitude questions developed to characterize a disease prevention model. Therefore, this domain mainly consists of questions regarding CVD’s risk factors. The practice domain was based on the preventive strategies for CVD.

The point to note is that we did not consider items regarding alcohol consumption since it remains one of the most challenging lifestyle behaviors with multiple determinants [17]. Although excessive alcohol consumption is a significant risk factor for many health problems such as cardiovascular diseases, light-to-moderate alcohol consumption has shown cardio-protective [18]. Besides, the definition of the dose of alcohol that protects from cardiovascular outcomes has been challenged [19]. Many studies have shown that religious and socio-cultural factors highly influence the acceptability of alcohol consumption behaviors [20]. Furthermore, alcohol consumption is linked to stigma and social embarrassment in Islamic countries [21]. On the other hand, one of the five essential characteristics of items required to ensure the quality of construct measurement recommended by Fowler is the willingness of respondents to provide the correct answers needed for the question at all times [22]. Thus, given the importance of reporting correct answers during research studies and the sensitive nature of such information in the culture of Islamic countries, asking about such sensitive information would not be acceptable.

Finally, the initial set of CVD-KAP29 consisted of 62 items were extracted according to the specialists’ responses and related literature available and were categorized into three subscales of knowledge, attitude, and practice on CVD risk factors, symptoms, and protective factors. Items on knowledge (items 1 to 30) were measured using a three-point rating scale in which interviewees were asked to mark their agreement with one of the possible responses of “2=Yes, 0=No, 1=I don’t know “. Items on attitude (31 to 48) were measured using a five-point Likert scale with possible responses of “1 = Strongly Disagree, 2 = Disagree, 3 = Neutral, 4 = Agree, 5 = Strongly Agree”. Practice items (49 to 62) were measured using a three-point rating scale of 0 to 2 depending on the response of each item. Furthermore, one more section for personal questions regarding age, gender, education, parity, occupation, marital status, residential area, and history of CVDs was added to the questionnaire.

### Phase II: content validity

To evaluate the content validity of the CVD-KAP29, we formed a multidisciplinary panel of 12 experts (Fig. 1). In step 1, five experts, including three cardiologists, one epidemiologist, and one health promotion expert, reviewed and commented on the initial questionnaire to assess qualitative content validity. In step 2, to evaluate quantitative content validity, the whole panel experts were requested to determine each item’s relevance, clarity, simplicity, and necessity. Then the content validity ratio (CVR) and content validity index (CVI) of each item was calculated. The CVR proposed by Lawshe measures the essentiality of each item. The formula for the CVR is CVR = (Ne – N/2)/(N/2), in which Ne is the number of experts indicating an item as “essential” and N is the total number of experts [23]. The CVR values can range from − 1 to + 1, indicating perfect disagreement and perfect agreement, respectively. Lawshe’s table was used to determine acceptable CVR values [23]. The CVI measures relevancy, clarity, and simplicity of each item and was calculated using the percentage of experts ranking each item as “relevant or very relevant/clear/simple” (rating 3 or 4). CVI values range from 0 to 1, in which a CVI > 0.79 representing the relevancy of the item, between 0.70 and 0.79 indicating that some revisions are needed for the item, and the value less than 0.70 showing the elimination of the item based on experts’ statements. Simultaneously, some questions in the general section were also reformulated during this phase.

### Phase III: face validity

Ten individuals with different sex, age, and education levels were asked to ascertain whether the items were understandable by checking the following options (good, moderate, or bad) to assess qualitative face validity and avoid misunderstanding. The item impact score was used to determine the quantitative face validity of each item. The impact score for each of the 29 items was calculated using a five-point Likert scale with potential answers from not important (score 1) to very important (score 5) and the formula of Frequency (%) × Importance (score 3 or 4). An impact score higher than 1.5 was considered for keeping the item for further analysis.

### Phase IV: construct validity

Exploratory Factor Analysis (EFA) with principal components method and varimax rotation was conducted to assess the multidimensional structure of the questionnaire for knowledge, attitude, and practice subscales. Kaiser-Meyer-Olkin (KMO) measurement of sampling adequacy (> 0.6) and Barlett’s test of sphericity (*p* < 0.001) were considered for acceptable factor analysis (Cerny and Kaiser, 1977). Factor loadings > 0.3 were supposed to be “meaningful”, and items with loadings below 0.3 were removed [24, 25]. A Confirmatory Factorial Analysis (CFA) was also performed to check whether data fit the structure model extracted by EFA. Chi-square less than three and root mean square error of approximation (RMSEA) less than 0.08 were considered as acceptable model fit indices and cut-off points of these indices for CFA.

### Phase V: reliability analysis

The test-retest reliability was assessed by intra-class correlation coefficients (ICCs). The questionnaires were completed by 50 individuals twice at an interval of 2 weeks. An ICC below 0.4 indicated poor reliability, 0.4 to 0.59 is fair, 0.6 to 0.74 is good, and 0.75 showed excellent reliability. We estimated the reliability of the CVD-KAP29 based on Cronbach’s α and McDonald’s ω [26].

Statistical analysis was performed using IBM SPSS 20.0 [27], LISREL 8.8, and MBESS Package [28] in R version 4.0.3 [29].

## Results

Figure 1 displays the consecutive steps of questionnaire development. A total of 300 (51.3% female) adults aged 21–74 completed the CVD-KAP29. The socio-demographic status of the participants is represented in Table 1. There was no statistical difference between urban and rural participants regarding age, sex, and marital status. A more proportion of urban participants had a university education and were employed (Table 1).

**Table 1:** Baseline characteristics of the participants (*n* = 300)

Variable		Total (%)	Residence		***p***-value*
			Urban (***n*** = 164)	Rural (***n*** = 133)	
**Age (Mean ± SD)**		39.72 ± 12.1	40.0 ± 12.8	40.1 ± 13.2	0.504
**Sex**	Male	146 (48.7)	87 (53.1)	57 (42.9)	0.081
	Female	154 (51.3)	77 (47.0)	76 (57.1)	
**Marital status**	Never married	51 (17.0)	30 (18.3)	21 (15.8)	
	Married	237 (79.0)	128 (78.1)	106 (79.7)	0.330
	Divorced	5 (1.7)	4 (2.4)	1 (0.75)	
	Widow	7 (2.3)	2 (1.2)	5 (3.8)	
**Education level**	Primary school	14 (4.7)	4 (2.4)	10 (7.5)	
	Under Diploma	52 (17.3)	25 (15.2)	27 (20.3)	< 0.001
	Diploma	102 (34.0)	44 (26.8)	56 (42.1)	
	University education	132 (44.0)	91 (55.5)	40 (30.1)	
**Occupation status**	Employed	87 (29.0)	52 (31.7)	35 (26.3)	
	Worker	22 (7.4)	17 (10.4)	3 (2.3)	
	Self-employment	79 (26.3)	40 (24.4)	38 (28.6)	0.012
	Student	6 (2.0)	3 (1.8)	3 (2.3)	
	Housekeeper	93 (31.0)	42 (25.6)	51 (38.4)	
	Retired	13 (4.3)	10 (6.1)	3 (2.3)	

* Independent t-test for continuous variables and chi-square or Fisher exact’s test for categorical variables

### Phases I, II, and III: item generation, questionnaire design, content validation, and face validity

Initially, a pool of 62 items was extracted according to the specialists’ responses in the interviews and related literature available. First, in the qualitative content validity, 32 items were removed due to repetitive versions of other items, confusing or irrelevant items based on the experts’ opinion. A satisfactory level of agreement was found among panelists on 30 remained items, and alterations were made to them to make them more precise and enhance clarity. Second, in the quantitative content validity, the CVR and CVI of 30 items of the questionnaire were 0.80 and 0.91, respectively, suggesting a good content validity of the remaining items (Table 2).

**Table 2:** Results of Content validity ratio, Content validity index, and Face validity of CVD-KAP29

Item Number	CVR	CVI (Simplicity)	CVI (Relevancy)	CVI (Clarity)	CVI (Total)	Impact score
**1**	0.87	1	1	1	1	2.7
**2**	1	0.95	0.95	0.90	0.93	3.4
**3**	0.87	1	1	1	1	3.6
**4**	0.87	0.90	1	0.90	0.93	3.8
**5**	1	1	1	1	1	3.7
**6**	0.87	0.94	1	0.86	0.93	3.6
**7**	0.87	0.96	0.88	0.94	0.93	3.1
**8**	0.73	0.87	0.87	0.87	0.87	3.2
**9**	1	1	1	1	1	3.3
**10**	0.73	0.84	0.95	0.84	0.87	3.6
**11**	0.87	0.88	0.97	0.93	0.93	3.3
**12**	0.73	0.88	0.90	0.84	0.87	3.7
**13**	0.86	0.92	1	0.92	0.94	3.8
**14**	0.63	0.94	0.94	0.94	0.94	3.1
**15**	0.88	1	1	1	1	3.5
**16**	0.88	0.92	1	0.92	0.94	3.4
**17**	0.63	0.94	0.94	0.94	0.94	3.6
**18**	0.75	0.84	0.95	0.84	0.88	3.8
**19**	0.75	0.88	0.90	0.86	0.88	3.4
**20**	0.63	0.78	0.88	0.78	0.81	2.9
**21**	0.75	0.94	0.96	0.92	0.94	3.2
**22**	0.63	0.86	0.95	0.84	0.88	3.2
**23**	0.73	0.84	0.95	0.84	0.87	3.1
**24**	0.87	0.93	0.93	0.93	0.93	3.2
**25**	0.87	0.92	0.97	0.90	0.93	3.4
**26**	0.73	0.92	0.97	0.90	0.93	3.1
**27**	0.73	0.87	0.87	0.87	0.87	3.2
**28**	0.73	0.88	0.90	0.84	0.87	3.4
**29**	0.87	0.88	0.90	0.84	0.87	3.8
**30**	0.60	0.80	0.80	0.80	0.80	2.7

Based on face validity results, all of the items were clear and easy to understand except one that needed to be revised. Furthermore, the impact scores showed that all the items had a score equal to or greater than 1.5, hence included in the questionnaire (Table 2).

### Phase IV: construct validity

In the exploratory factor analysis, the Kaiser-Meyer-Olkin test (0.864) and Bartlett’s test of sphericity (chi-squared, df = 3659.663, 435; *p*-value < 0.001) showed that items met the criteria required for factor analysis; scree plot revealed eight factors with eigenvalues above 1. The total variance explained by these nine factors was 63.3%, and the final analysis was repeated with a four-factor solution using a varimax rotation.

Table 3 presents the items along with their factor loadings for knowledge, attitude, and practice subscales. Only q30 had a factor loading < 0.3, so 29 items were retained in the final model. Overall, the total percentage of variance was 48.43; the percentage of variance explained by each subscale was 17.04 for knowledge, 19.51 for attitude, 5.50 for physical activity-related behavior, and 6.33 for nutrition & smoking-related behaviors. In the final model, 29 items were retained from the initial 30 items. Based on the results of the CFA model with 29 remaining items in four subscales, root mean square error of approximation (RMSEA) = 0.068, comparative fit index (CFI) = 0.94, the goodness of fit index (GFI) = 0.83, normed fit index (NFI) = 0.90, and incremental fit index (IFI) = 0.9, indicating acceptable model fit indices.

**Table 3:** Factor loading matrix of CVD-KAP29

Items	Factor loading			
	Knowledge	Attitude	Physical activity behaviors	Nutrition & smoking behaviors
1: Cardiovascular diseases are the leading cause of death in Iran.	**.440**	.187	−.026	.177
2: Physical activity can prevent cardiovascular disease.	**.636**	.137	−.006	.045
3: Daily eating of fruits and vegetables has a beneficial effect on cardiovascular health.	**.674**	−.042	.073	−.081
4: The history of cardiovascular disease in the family (father, mother, sister, or brother) can increase the risk of cardiovascular disease.	**.575**	.115	.014	.039
5: There is a higher risk of cardiovascular disease in people who are overweight or obese.	**.586**	.023	.009	−.183
6: Using tobacco (cigarettes, hookah, pipe, etc.) can increase the risk of cardiovascular disease.	**.738**	.057	−.068	−.092
7: Consumption of salty and canned foods increases the risk of rising blood pressure.	**.710**	.137	−.017	−.014
8: Controlling blood glucose and the prevention of diabetes can reduce the risk of cardiovascular complications.	**.644**	.212	.069	.029
9: Controlling high blood pressure is vital to prevent myocardial infarction.	**.746**	.144	−.159	−.047
10: Feeling of pain, pressure, or burning in the chest can be a symptom of a heart attack.	**.676**	.005	−.069	.094
11: Feeling of pain or sudden discomfort in the jaw, neck, between the two scapulas, shoulders, or arms and stomach area can be a symptom of a heart attack.	**.595**	.135	.044	.235
12: Sudden numbness or weakness in the face, arms, or legs muscles can be signs of a stroke.	**.594**	.198	.105	.168
13: I believe that I should have physical activity to have a healthy life.	.068	**.816**	.077	.037
14: I believe that I should try to walk to go to nearer destinations instead of going by taxi or bus.	.048	**.706**	.042	.083
15: I believe that using any tobacco (cigarette, hookah, pipe, etc.) is harmful to health.	.044	**.721**	.076	.194
16: I believe that having an appropriate weight (not overweight or obesity) helps keep me healthy.	.216	**.551**	.022	.057
17: I believe that I must consume less fatty foods to maintain health.	.114	**.820**	.007	.013
18: I believe that daily consumption of 2 to 4 units of fruit and 3 to 5 units of raw or cooked vegetables is beneficial for my health.	.107	**.820**	−.049	−.006
19: I believe that uncontrolled blood glucose in diabetic patients can cause myocardial infarction.	.202	**.685**	−.031	.191
22: I believe that I should control my stress and mental pressure to prevent myocardial infarction.	.146	**.796**	−.024	.092
21: I believe that I should consume less salt to prevent high blood pressure.	.159	**.799**	−.145	.018
22: I believe that consuming fish meat at least two times a week is beneficial for cardiovascular health.	.120	**.688**	−.092	.042
23: Do you have intense physical activity such as running, carrying heavy loads, drilling, and ... at least 30 min a day during the week?	.009	−.013	**.849**	.000
24: Do you have moderate physical activity such as fast walking or carrying light loads at least 30 min a day during the week?	.046	.047	**.859**	.012
25: Do you have 2 to 4 units of fruit and 3 to 5 units of raw or cooked vegetables in your daily diet plan?	.040	.022	−.085	**.727**
26: What oil do you usually use more for cooking?	.062	−.047	.133	**.647**
27: What oil do you use more for frying foods?	−.043	.141	.030	**.443**
28: Do you add salt to your food at the table?	.079	.175	−.135	**.530**
29: Do you currently use any tobacco products (cigarette, hookah, pipe, etc.)?	−.015	.031	−.001	**.403**
30: Do you take your medications according to your doctor’s prescription if you have any chronic illness such as cardiovascular disease, diabetes, high blood pressure, or high blood lipids?	.086	.191	−.221	.193
% of explained variance	17.049	19.519	5.527	6.334

Extraction Method: Principal Component Analysis.

Rotation Method: Varimax.

Kaiser-Meyer-Olkin Measure of Sampling Adequacy. 0.864

Bartlett’s Test of Sphericity *p* < 0.0001 (Approx. Chi-Square: 3659.663, df:435)

**Fig. 2:**
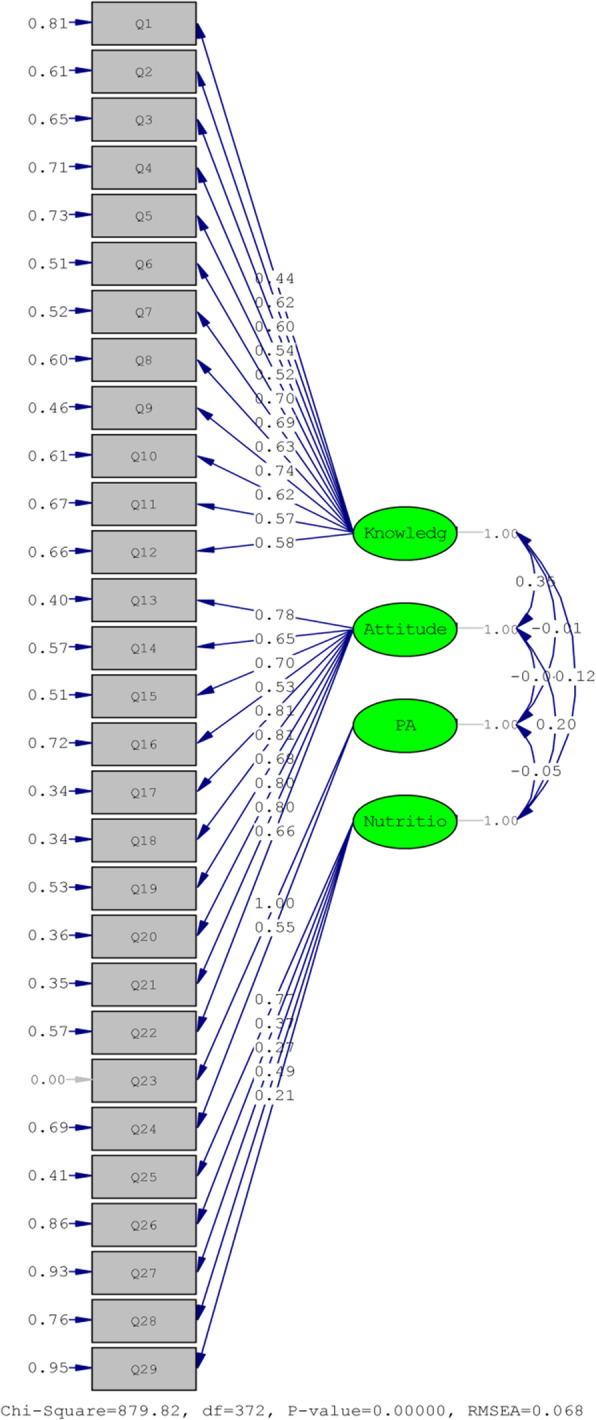
Standardized factor loadings of the measurement model of CVD-KAP29. Four latent constructs and 29 observed items were included in Confirmatory Factor Analysis (CFA)

Figure 2 shows the hypothetical structure of the CFA model with 4 factors and 29 finalized items. Standardized factor loadings are shown at top of the paths. Moreover, the correlation between subscales and its significance criteria (T-values) was significant on the paths. For the “knowledge” subscale, the maximum and minimum loadings were regarding Q9 (λ = 0.74, T = 14.18) and Q1 (λ = 0.44, T = 7.52), respectively. For the “attitude” subscale the maximum and minimum loadings were regarding Q17 (λ = 0.81, T = 16.76) and Q16 (λ = 0.53, T = 9.16), respectively, and for the nutrition & smoking behaviors, the maximum and minimum loadings were regarding Q25 (λ = 0.77, T = 8.61) and Q29 (λ = 0.21, T = 3.05), respectively.

### Phase V: reliability analysis

Intra-class correlation coefficients for knowledge, attitude, and practice subscales were 0.85, 0.95, 0.57, and 0.36, respectively (*p* < 0.001), representing good-to-excellent test-retest reliability in knowledge and attitude subscales and poor reliability in the practice subscale. Generally, Cronbach’s alpha coefficient for each subscale was 0.856 for knowledge, 0.915 for attitude, 0.711 for physical activity-related behaviors, and 0.509 for nutrition & smoking behaviors. McDonald’s ω for the CVD-KAP29 was also satisfactory, with values of 0.859 for knowledge, 0.918 for attitude, 0.715 for physical activity-related behaviors, and 0.576 for nutrition & smoking behaviors.

### Final questionnaire

At the end of the above steps, the final version of the questionnaire, known as CVD-KAP29, consisted of five sections; section 1, comprising 12 items related to personal questions; section 2, 12 valid and reliable items related to knowledge in which the total raw scores ranged from 0 to 24; section 3 had ten items related to the attitude in which total raw scores of attitude ranged from 10 to 50; section 4, included two items related to physical activity behaviors and finally section 5, including five items related to nutrition & smoking behaviors.

## Discussion

This study was carried out to design and measure the reliability and validity of a questionnaire for assessing the knowledge, attitudes, and practice regarding risk factors, symptoms, and protective factors of cardiovascular disease among Iranian adults. In this assessment study, a sequence of steps was taken to improve the initial tool and determine its validity and reliability. The final questionnaire consisted of 29 statements (CVD-KAP29), and the results confirmed this developed questionnaire’s good reliability and validity.

It has been suggested that without knowing the validity and reliability of a scale, it is impossible to see if it measures what it claims to measure, even for knowledge and behavior subscales [30]. Furthermore, it helps researchers know about the representativeness of the items for each domain and points of ambiguity and accuracy [30, 31]. In the current study, the psychometric properties of CVD-KAP29 could not be compared to previous studies on the knowledge, attitudes, and practices of CVDs due to inadequate information and unclearness regarding the questionnaire development and validation processes reported in the studies [32–37].

For the determination of content validity, we used the Lawshe approach [38]. The experts ‘panel found an acceptable level of agreement (CVI = 0.89 and CVR = 0.86), indicating a good content validity. In determining face validity, clarity, and understanding of items by the target group are vital; adults well understood the majority of the items developed in the KAP-CVD. The importance of face validity in determining the appropriateness of a questionnaire has been emphasized in several questionnaires designed for different targets [39, 40].

While the items comprising the CVD-KAP29 scale were initially selected to cluster on three dimensions (Knowledge, Attitude, and Practice), in the evaluation of construct validity, the EFA resulted in a better-fitting four-factor model (two factors for the practice subscale), with better reliability and internal consistency than the three-factor model. Furthermore, one practice item presented lower factor loadings and was excluded. Hereof, the practice subscale was separated into two factors: (1) physical activity practices that contained questions related to physical activity and (2) Nutrition & Smoking practices, which included questions related to nutrition behavior and the use of tobacco products.

While the EFA is used to establish the most appropriate factor structure of the questionnaire based on the pattern-linearity of the factor loadings, the application of CFA is worthwhile to complement the construct validity by confirming the factorial structure and their respective items identified in the EFA [41–44]. In the current study, the four-factor structure was established by CFA, indicating the satisfactory fitting of the suggested models.

In the present study, both Cronbach’s alpha and McDonald’s ω reliability coefficients were ranged from 0.51–0.92, confirming the internal consistency and reliability of CVD-KAP29. The test-retest reliability of the CVD-KAP29 was determined by the intra-class correlation coefficient (ICC) between the first and second administrations of the questionnaire with a two-week interval, a duration considered appropriate, as it is neither too short to remember the responses nor is too long to change their knowledge, attitude and practice [45, 46]. Most correlations of the subscales in the CVD-KAP29 were acceptable, ranging from low reliability for practice to excellent for attitude (0.36 ~ 0.95). As the practice is not a feature that tends to be constant over time, the reliability was found to be low for that; knowledge and attitude, in particular, can change the individuals’ behavior [5]. Individuals’ practices also depend on other factors, including physical, sociocultural, economic environments, and social media and advertising [5].

One strength of this study was developing and evaluating the validity and reliability of a questionnaire for the general population related to all aspects of cardiovascular disease. However, some limitations and recommendations should be addressed. Participants were selected from only one city in Iran. Besides, this questionnaire was validated among Tehranian adults, and due to the different environment and culture may not be appropriate for populations in other Iranian provinces and countries. Hence, it is recommended that researchers validate and modify the CVD-KAP29 among other people and use this questionnaire as baseline data for future prevention interventions.

## Conclusion

This study provides evidence of the validity of the CVD-KAP29, which can hence serve as an essential tool to evaluate individuals’ knowledge, attitude, and practices on risk factors, symptoms, and protective factors of cardiovascular disease among the general population. This information would be valuable in developing and implementing targeted educational programs to prevent cardiovascular diseases in general populations. Further studies are recommended in different urban and rural populations.

## Data Availability

The datasets used during the current study are available from the corresponding author on reasonable request.

## Abbreviations

KAP: Knowledge, Attitude, and Practice
CVD: Cardiovascular Diseases
CVD-KAP29: Knowledge, Attitudes and Practice questionnaire for Cardiovascular Diseases
GBD: Global Burden of Disease
CVR: Content Validity Ratio
CVI: Content Validity Index
EFA: Exploratory Factor Analysis
KMO: Kaiser-Meyer-Olkin
RMSEA: Root Mean Square Error of Approximation
ICCs: Intra-class Correlation Coefficients
